# Robustness Evaluation of the Open Source Product Community Network Considering Different Influential Nodes

**DOI:** 10.3390/e24101355

**Published:** 2022-09-24

**Authors:** Hongli Zhou, Siqing You, Mingxuan Yang

**Affiliations:** 1 School of Information, Beijing Wuzi University, Beijing 101149, China; 2 School of Management, University of Bristol, Bristol BS8 1TH, UK

**Keywords:** open source product community network, robustness, structural hole, opinion leader

## Abstract

With the rapid development of Internet technology, the innovative value and importance of the open source product community (OSPC) is becoming increasingly significant. Ensuring high robustness is essential to the stable development of OSPC with open characteristics. In robustness analysis, degree and betweenness are traditionally used to evaluate the importance of nodes. However, these two indexes are disabled to comprehensively evaluate the influential nodes in the community network. Furthermore, influential users have many followers. The effect of irrational following behavior on network robustness is also worth investigating. To solve these problems, we built a typical OSPC network using a complex network modeling method, analyzed its structural characteristics and proposed an improved method to identify influential nodes by integrating the network topology characteristics indexes. We then proposed a model containing a variety of relevant node loss strategies to simulate the changes in robustness of the OSPC network. The results showed that the proposed method can better distinguish the influential nodes in the network. Furthermore, the network’s robustness will be greatly damaged under the node loss strategies considering the influential node loss (i.e., structural hole node loss and opinion leader node loss), and the following effect can greatly change the network robustness. The results verified the feasibility and effectiveness of the proposed robustness analysis model and indexes.

## 1. Introduction

Open source design (OSD) is widely recognized and welcomed by the public. Enthusiasts from all over the world use open source communities (OSCs) to put forward their product design ideas and to carry out collaborative innovation. Under this bottom–up organizational structure, many high-quality, low-cost products have been produced, and open source product communities (OSPCs) often have rich knowledge resources and broad market values [1,2]. At present, successful OSPC products, such as Linux operating system, Mozilla Firefox, Python and Apache web server are developed by large groups of volunteers, who provide expertise from a multitude of professional backgrounds at a very low cost. These software systems have a high robustness and adaptability. The famous OSD platform, OpenIDEO, solves real-world problems by exploring perspectives, tools, and ideas from the edges of social impact and innovation. As the proponents and designers of product ideas, users are essential to an OSC [3], and their continuous participation is the driving force of an OSC’s development. This is particularly true of important contributors, who undertake most of the tasks of the project and are the promoters of its success [4,5,6]. As such, attracting more users and accessing users’ full expertise and wisdom are crucial for the long-term development of an OSC. However, there exists a fierce competitive relationship between the same types of OSC to win users. Additionally, community managers can pay too much attention to the short-term external scale of the community, ignoring the construction of its internal management mechanism. This leads to frequent changes and even a loss of users, which can cause the failure of the cooperative relationships between the community’s remaining users [7,8]. In particular, a loss of influential users may cut off the communication channel of community information, causing panic among other users, aggravating user loss and leading to an interruption of the project, which puts the community at risk of decline [9]. Robustness represents the ability of a system to maintain its functions or properties under interference [10]. When an OSC faces a loss of influential users, an analysis of the robustness of the community can be carried out to evaluate its risk resistance capacity. By detecting the loss pattern, degree of loss and identifying which users affect the robustness of the community, community managers can formulate targeted and timely strategies to deal with the loss. This shows that research on the robustness of the OSPC considering influential user loss has important practical significance.

The robustness of complex networks has been studied from various perspectives with great results. Many studies focus on the robustness of complex networks in real systems (e.g., supply chains, transportation, power networks, IT infrastructure, biological network etc.) [11,12,13,14,15,16], and many are on virtual or online social systems (e.g., the cooperation network, the online social network, the Email communication network, the Internet etc.) [17,18,19,20]. With the rapid development of OSCs, research on their robustness has become increasingly important. For example, Lei et al. [21] constructed a weighted network based on the semantic analysis of collaborative behavior to study the robustness of the open source project network. Zhou et al. [22] designed different node attack strategies and studied the robustness of the open source product community in different periods of evolution in a dynamic environment. Tanaka et al. [23] demonstrated that the nonlinear dynamics of heterogeneously connected networks can be highly vulnerable to the failure of low-degree nodes in complex networks. Tang and Liao [24] studied the robustness of the regional collaborative innovation network by removing nodes with high betweenness. Note that existing research on network robustness usually uses the degree and betweenness of nodes in the network to measure the importance of nodes as the basis for the selection of lost nodes. However, in their study of complex networks, Kitsak et al. [25] found that simply relying on the node degree and betweenness cannot accurately describe the influence of a node in the network, and the node importance also depends on its position within the network. Burt [26] found that some structural hole nodes in complex networks play the role of “connector”. These nodes can obtain more critical information because they are in an advantageous location in the network. They bring benefits, such as the promotion of position and improvement of reputation to participants, and they can affect the relationship between nodes in the network and even control the dissemination of information. Han et al. [27] found that although the core node is very important in the process of network topic dissemination, the node in the structural hole makes up for the limitations of information dissemination among different groups and plays an important role in information diffusion. In addition, structural hole nodes are also used as an index to measure the importance of nodes in the network. For example, Sotoodeh and Falahrad [28] took the node degree ratio of structural holes into account when mining influential nodes in complex networks. Liu et al. [29] proposed a node information control ability index according to the structural hole characteristics of nodes to identify important nodes that affect security in complex networks. In identifying structural hole nodes, Ruan et al. [30] presented an effective method based on local characteristics to identify structural hole nodes that play important roles in maintaining network connectivity. Ding et al. [31] provided an improved method to detect key nodes that occupy structural holes according to the features of a social network site. This considers the degree of nodes and the topological structure of their neighbor nodes.

In addition to the structural hole node, scholars also discovered a special node in the network environment that can dominate the dissemination direction of network information and promote the dynamic change of network resources. This is known as the “opinion leader” node. This kind of node conforms to the classical structural hole theory. Its position in the network is vital, as it not only affects personal information and power, but also controls the resource flow of the whole network [32]. The influence of opinion leaders can even guide the trend of network public opinion and affect the emotional attitudes or behaviors of others [33]. When investigating following behavior, Blincoe et al. [34] found that users who are both very popular and very active have an influence on their followers. Further, van Eck [35] suggested that opinion leaders are influential, not only because of the number of relationships they have, but also because they are more innovative, have better product judgment. Therefore, when studying the robustness of a network, it is necessary to identify the opinion leader nodes in the network. In the field of network public opinion, scholars have studied the identification and communication characteristics of opinion leaders. For instance, Zhang et al. [36] created a network opinion leader recognition method based on relational data, and designed an opinion leader discovery module based on Markov logic networks. Ye and Du [37] proposed a method that incorporated sentiment analysis to mine opinion leaders and find dominant users. Ma and Liu [38] proposed a SuperedgeRank algorithm for opinion leader identification based on supernetwork theory, which combined network topology analysis and text mining. The research stated above proves that structural hole nodes and opinion leader nodes play a significant role in the information flow and knowledge diffusion in the network. Thus, they are influential nodes that cannot be ignored. However, little is known about the specific impact of the loss of structural hole nodes and opinion leader nodes on the robustness of complex networks. The relationship between structural hole nodes, opinion leader nodes and common nodes in the network also needs further study.

As an OSC evolves, the robustness of its network plays a vital role in the survival and development of the community [21,22,39]. However, in the OSPC, managers have not paid enough attention to how important structural hole nodes and opinion leader nodes are to network robustness. To accurately describe the role of these influential nodes and how they affect the OSPC network’s robustness, this paper first accurately identifies these nodes, then proposes a robustness analysis model and indexes to carry out a robustness simulation experiment.

The remainder of this paper is organized as follows. In Section 2, we introduce the network construction method and analyze the network topology parameters and network characteristics. In Section 3, we describe the methods used to identify and mine the structural hole nodes and opinion leader nodes. In Section 4, based on the realistic behavior of the loss of influential nodes, we propose the failure strategies of these nodes and introduce the indexes for the network’s robustness evaluation. We carry out the robustness simulation experiment and discussions in Section 5. Finally, our conclusions and outlook are given in Section 6.

## 2. Topological Characteristics of an Open Source Product Community Network

### 2.1. Data Sources

As a new knowledge production mode, OSD makes full use of the knowledge and creativity of internet users, and an increasing number of people are participating in design through OSCs. We selected the Local Motors community as our research object. This community creates a new mode where users can take part in the online design and offline production of personalized vehicles. Anyone, not just those from within the organization, can freely join the community—initiated challenges and propose design solutions. The community receives and votes on the most feasible solution. A prototype of design scheme is processed, tested and iterated in a collaboration cycle, and the product is finally made according to the customer’s needs.

This community has a relatively mature operation system and a large collection of projects and users. We obtain the relevant data of 11 projects that have a large number of participants and a high degree of project completion in the Local Motors community from 20 May 2008 to 30 November 2016. The preliminary analysis of the data shows that these projects were established at various time points, and their completion progress also varies. Furthermore, the project attributes, user attributes, user communication and collaboration, and other information in the original data were preprocessed, and information irrelevant to project innovation and product development was filtered out. There are 1428 users working on these 11 projects, with 8955 communication events relating to knowledge collaboration.

### 2.2. Network Construction and Topological Characteristics

In an OSPC, project initiators and participants (collectively referred to as users) can freely express their views and ideas. User behaviors, such as evaluation, suggestion, likes and sharing make the community show macro-complex network characteristics. Accordingly, based on the complex network theory, this paper constructs a directed weighted knowledge collaboration network (KCN), where nodes represent users who contribute and disseminate knowledge, and edges represent the collaboration behavior between nodes. The network model is defined as *G* = (*V*,*E*,*W*), where *V* is the set of nodes in the community, *E* is the set of edges, which indicates that there is an interactive relationship between node *i* and node *j*, and *W* is the weight of node *i*. The network topology parameters and network characteristics are calculated according to the social network analysis method, as shown in Table 1.

As shown in Table 1, the average degree of the KCN is 6.27, indicating that users collaborate and communicate frequently. The average path length is 3.04, indicating that any two users can contact each other through an average of three paths. A shorter average path length helps nodes acquire non-redundant information, improving the efficiency of information dissemination in the KCN. The clustering coefficient is 0.53, illustrating that users tend to collaborate in groups. This matches the pattern of user participation in projects in the community. The analysis of the KCN’s characteristics shows that the network has obvious small-world and scale-free characteristics. The small-world characteristic proves that there are some opinion leaders who have influence in the network, and the scale-free characteristic shows that the network is highly robust against random attacks.

**Figure 1:**
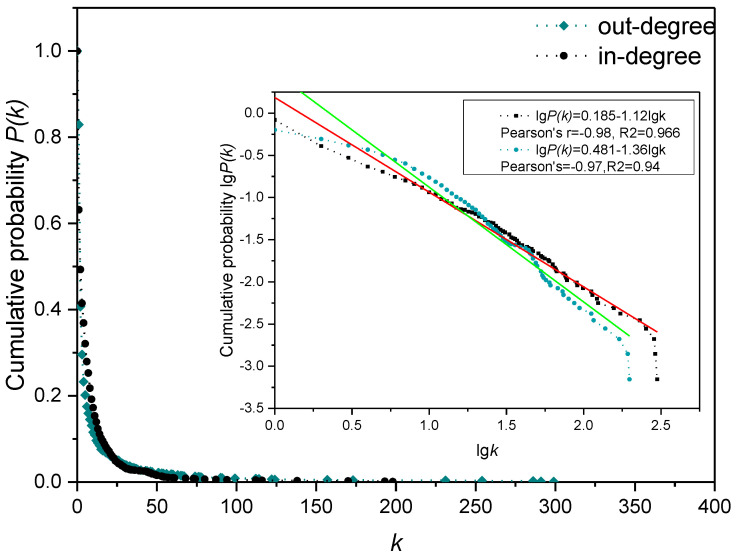
Out-degree and in-degree distribution of the knowledge collaboration network.

**Figure 2:**
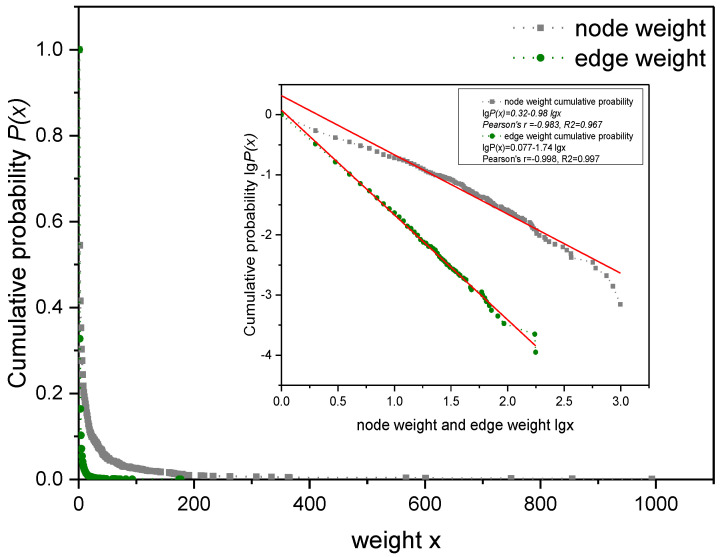
Node weight and edge weight distribution of the knowledge collaboration network.

As indicated in Figure 1 and Figure 2, the out-degree, in-degree, node weight and edge weight of the node relationship in the directed weighted knowledge collaboration network all obey the power-law distribution. This shows that the behavior of community users in the process of knowledge collaboration has greater heterogeneity, that is, a few users participate in more projects and conduct a lot of knowledge collaboration with other users, while more users only participate in few projects and have less knowledge collaboration with other users. Therefore, identifying these influential nodes is critical to the effective operation of the open source product community.

## 3. Identification of Influential Nodes

### 3.1. The Identification of Structural Hole Nodes in Network

In complex networks, the importance of a node depends not only on the number and quality of other nodes connected to the node, but also on its location within the network. In the Local Motors community, User A might participate in multiple projects simultaneously, and they might cooperate with User B, User C, User D, etc. in these projects. However, there might also be no communication and collaboration among Users B, C and D, so User A plays a key “bridging” role in knowledge dissemination and information flow in the community. According to Burt’s structural hole theory [26], User A is a structural hole node in the network. The node can obtain more critical information in a timely manner, thereby affecting or even controlling the dissemination and diffusion of information throughout the network. The nodes on both sides of the structural hole form knowledge complementarity through indirect connection, and the structural hole node obtains large amounts of non-redundant knowledge, further promoting the knowledge integration and improvement of the node itself. Therefore, compared with other nodes, structural hole nodes will gain a greater competitive advantage in the process of network evolution and are more likely to become opinion leaders in the network.

In related research on the identification of structural hole nodes [26], Burt proposed four structural hole analysis indexes: effective scale, efficiency, degree of hierarchy and degree of constraint. The constraint degree, which describes the ability of nodes to utilize structural holes in the network, is the most used of these indexes. The smaller the coefficient, the greater the degree of structural holes, the more important the position of the node, and the more able the node is to occupy and span the structural hole. Therefore, we take the constraint degree as the main reference index to identify the structural hole nodes in the network:

(1) $$c_{ij}={\left(p_{ij}+\underset{q}{\sum }p_{iq}p_{qj}\right)}^{2}$$

where q≠i,j, node q is the common neighbor of nodes i and j, $p_{ij}$ is the proportion of the total energy that node i invests in maintaining its neighborly relationship with node j, and $p_{iq}$ and $p_{qj}$ are the respective proportions of the total energy that nodes i and j invest in maintaining their relationships with their common neighbor q. The calculation methods of $p_{iq}$ and $p_{qj}$ are similar to that of $p_{ij}$, which is as follows:

(2) $$p_{ij}=z_{ij}/\underset{j\in \Gamma \left(i\right)}{\sum }z_{ij}$$

where $z_{ij}=\{\begin{array}{c} 1,\;i\;\mathrm{to}\;j\;\mathrm{have}\;a\;\mathrm{link}\\ 0,\;i\;\mathrm{to}\;j\;\mathrm{have}\;\mathrm{no}\;\mathrm{link} \end{array}$.

The total constraint degree of nodes is:

(3) $$C_{i}=\underset{j}{\sum }c_{ij}$$

Accordingly, the constraint degree of each node in the network can be calculated. The top ten nodes based on the constraint degree index are shown in Table 2.

### 3.2. The Identification of Opinion Leaders in the Network

Like other community networks, OSC networks have opinion leader nodes that dominate the direction of information dissemination, public opinion flow, opinion formation and evolution. In existing research, there are various methods to help identify opinion leader nodes in networks. However, few methods exist on how to identify opinion leaders in an OSPC, which focuses on knowledge sharing and collaborative innovation. Based on existing research, and according to the characteristics of the OSPC, this paper adopts the comprehensive evaluation method of node importance of multi-attribute decision-making indexes to systematically identify and evaluate the opinion leader nodes in the network [41]. Based on TOPSIS (technology for order preference by similarity to an ideal solution) [42], the multi-attribute decision-making method takes multiple indicators (e.g., degree centrality, closeness centrality, betweenness, structural hole and the clustering coefficient of a single node in the network) as a comprehensive evaluation method to determine the importance of nodes in the network.

Specifically, for the existing N nodes in the network, $A=\left\{A_{1},\ldots ,A_{N}\right\}$ is used to represent the decision set of each node. If there are m indicators to evaluate the importance of each node, the index attribute set is recorded as $E=\left\{E_{1},\ldots ,E_{m}\right\}$. Then, the decision value of the j-th index of the i-th node is recorded as $A_{i}\left(E_{j}\right),i=1,\ldots ,N;j=1,\ldots m$. The decision matrix, composed of nodes and their index attributes, is denoted as *P*, and is expressed as follows:

(4) $$P=\left(\begin{array}{ccc} A_{1}\left(E_{1}\right) & \cdots & A_{1}\left(E_{m}\right)\\ \vdots & \ddots & \vdots \\ A_{N}\left(E_{1}\right) & \cdots & A_{N}\left(E_{m}\right) \end{array}\right)$$

Each node index has a different meaning; therefore, to uniformly measure the size of each index, the index matrix is standardized and the normalized decision matrix is recorded as $R={\left(r_{ij}\right)}_{N\times m}$.

Assuming that the weight of the j-th index of the node is $w_{j}=(j=1,\ldots ,m,\sum w_{j}=1)$, it forms a weighted normalization matrix *Y* with the decision matrix *R*:

(5) $$Y=(y_{ij})=\left(w_{j}r_{ij}\right)=\left(\begin{array}{ccc} w_{1}r_{11} & \cdots & w_{m}r_{1m}\\ \vdots & \ddots & \vdots \\ w_{1}r_{N1} & \cdots & w_{m}r_{Nm} \end{array}\right)$$

Further, the positive ideal decision node $A^{+}$ and the negative ideal decision node $A^{-}$ are determined by the weighted normalization matrix Y, and $A^{+}$ and $A^{-}$ and are respectively expressed as:

(6) $$A^{+}=\left\{\underset{i\in L}{\max}\left(y_{i1},\ldots ,y_{im}\right)\right\}=\left\{y_{1}^{max},\ldots ,y_{m}^{max}\right\}$$

and

(7) $$A^{-}=\left\{\underset{i\in L}{\min}\left(y_{i1},\ldots ,y_{im}\right)\right\}=\left\{y_{1}^{min},\ldots ,y_{m}^{min}\right\}$$

where, L={1,…,N}.

The distances $B_{i}^{+}$ and $B_{i}^{-}$ are respectively calculated from each node to the positive ideal decision node $A^{+}$ and the negative ideal decision node $A^{-}$:

(8) $$B_{i}^{+}={\left[\overset{m}{\underset{j=1}{\sum }}{\left(y_{ij}-y_{j}^{max}\right)}^{2}\right]}^{1/2}$$

and

(9) $$B_{i}^{-}={\left[\overset{m}{\underset{j=1}{\sum }}{\left(y_{ij}-y_{j}^{min}\right)}^{2}\right]}^{1/2}$$

Finally, the closeness $O_{i}$ of each decision-making scheme is obtained, where the larger the value of $O_{i}$, the more important the node is in the network:

(10) $$O_{i}=\frac{B_{i}^{-}}{\left(B_{i}^{-}+B_{i}^{+}\right)},0\leq O_{i}\leq 1$$

To effectively evaluate the importance of nodes, this paper selects four indexes that can comprehensively reflect the influence of nodes in the network according to the characteristics of the KCN of the Local Motors community: node degree centrality, betweenness centrality, proximity centrality and structural hole.

Using this method, the node with the closeness value top-k is calculated, which is the opinion leader with the greatest influence in the OSPC. Table 3 lists the top ten opinion leaders.

Comparing Table 2 and Table 3 shows that, of the top five opinion leaders, two nodes belong to structural hole nodes at the same time. Further, of the top ten opinion leaders, seven nodes belong to structural hole nodes at the same time. This proves that structural holes are a key indicator for measuring important influential nodes in the network. Like nodes with large degree and betweenness, structural hole nodes are key nodes that a community manager should pay attention to. In addition, the comparative results show that with the evolution of the community, the structural hole nodes occupying the dominant position can grow into the opinion leaders of the community with a high probability.

## 4. Description of Robustness Analysis Method

### 4.1. Description of Node Loss Strategies

The interference and risks an OSPC faces usually come from users voluntarily leaving the community—known as “loss of users”. In the real community, the loss of users includes random natural loss and subjective deliberate loss. Random loss manifests as an irregular loss of nodes in the network (i.e., both the users who leave the community and their loss patterns are random), and deliberate loss refers to when users leave the community after being attracted by other communities or being dissatisfied with the project level and management mechanism of their community, which manifests as a regular loss of nodes in the network. The deliberate loss of users can be divided into collective loss and successive loss within a set period of time. To study the impact on the robustness of the community when influential nodes such as structural hole nodes and opinion leaders leave the community, both collectively and successively, we designed five node loss strategies, as shown in Table 4.

### 4.2. Network Robustness Evaluation Indexes

When the network is attacked, how to quantitatively describe the changes of network structure and performance is essential to robustness research. A KCN is defined here as robust, allowing users to participate in all projects and collaborate with other users in the community, even during the disruption of one of the projects. This feature is directly associated to the size of the largest connected component (LCC) of the network. The change of the LCC can reflect the connectivity and collapse of the network. The relative size of the LCC reflects the degree of knowledge cooperation among users in the network. In mathematical terms, the relative size of the LCC of the KCN is:

(11) $$S=\frac{N^{\prime }}{N}$$

where $N^{\prime }$ is the number of nodes in the network’s LCC, and N is the total number of nodes in the KCN. The parameter S is contained in the interval 0≤S≤1, and is a measure for robustness: a smaller S is associated with a fragile network and a larger S is associated with a robust network. When S is 0, users in the community complete the tasks independently; thus, the knowledge collaboration of the project is at its worst. When S is 1, all users carry out various forms of knowledge collaboration; thus, the knowledge collaboration of the project is at its best.

When node loss occurs in the network, the edges between the nodes disappear, and the propagation path and efficiency of information in the network are affected. Due to the small-world property of the OSPC, network efficiency can be used to measure the efficiency of information exchanges over the network. When studying the impact of node loss on a network’s characteristics, the network may be disconnected. Since network efficiency can be extended to a disconnected network, it becomes a better statistical measure with which to evaluate the robustness of the network, both before and after node loss.

Network efficiency is defined as:

(12) $${Ø}_{G}=\frac{1}{N\left(N-1\right)}\underset{i\neq j}{\sum }\frac{1}{d_{ij}}$$

where N represents the total number of nodes in the network, and $d_{\mathrm{ij}}$ represents the length of the shortest path between node *i* and node *j*. It is assumed that the efficiency between nodes *i* and *j* is inversely proportional to the shortest path, that is, $\varepsilon_{\mathrm{ij}}=1/d_{\mathrm{ij}}$. With this definition, when there is no path between node *i* and node *j*, $d_{\mathrm{ij}}=+\infty$, then $\varepsilon_{\mathrm{ij}}=0$, so ${Ø}_{G}\in \left[0,\;1\right]$. The global efficiency of the network is defined as the average efficiency of all node pairs.

## 5. Results and Analysis

Based on our constructed node loss strategies and the robustness evaluation indexes from Section 4, we simulated the robustness characteristics of influential nodes and random loss nodes under the strategies of collective and successive loss. We further simulated the impact of nodes on network robustness under the following effect.

### 5.1. Robustness of the Network under the Collective Node Loss Strategies

**Figure 3:**
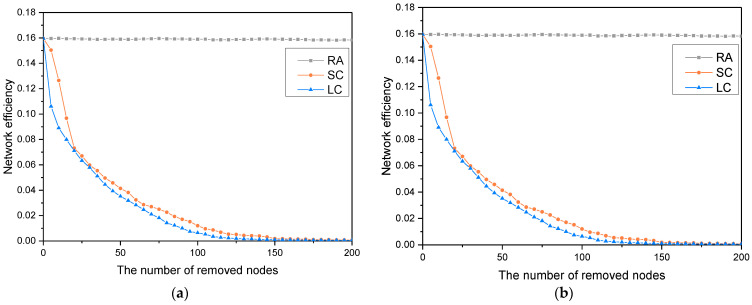
Comparative results of network robustness under collective node loss strategies. (**a**) changes in the relative size of the largest connected component; and (**b**), changes in network efficiency.

Figure 3 shows that with the relative size of the LCC and the network efficiency in the SC and LC, node loss strategies are significantly lower than in the RA node loss strategy. This shows that the robustness of the KCN in the face of a collective loss of structural hole nodes and opinion leader nodes is significantly lower than that in the case of a random loss of nodes. That is, the OSPC is highly robust against random failures, but is extremely fragile to deliberate attacks. This is consistent with the scale-free characteristics of the network. It also proves that the influential nodes (i.e., the structural hole nodes and the opinion leader nodes) play an important role in maintaining the stability of the community. With the large-scale collective loss of structural hole nodes and opinion leader nodes, network robustness rapidly decreases. Further, the robustness of the network in the LC loss strategy is lower than that in the SC loss strategy, indicating that the collective loss of opinion leader nodes is more destructive to the network, which also verifies the leadership position of opinion leaders in the network.

In addition, when 110 influential nodes are lost, the relative size of the LCC in the SC and LC strategies decreases by 74.8% and 96.7%, respectively, and the network efficiency decreases by 94.6% and 97.8%, respectively. However, when 150 influential nodes are lost, the relative size of the LCC in the SC and LC strategies decreases by 97.3% and 98%, respectively, and the network efficiency decreases by 98.8% and 99.4%, respectively. At this point, the network robustness tends to be close to 0. This shows that the robustness of the KCN depends primarily on these 150 influential nodes. When evaluating users, community managers should focus on these influential nodes, and they should formulate reasonable strategies to prevent their collective loss.

### 5.2. Robustness of the Network under Successive Node Loss Strategies

**Figure 4:**
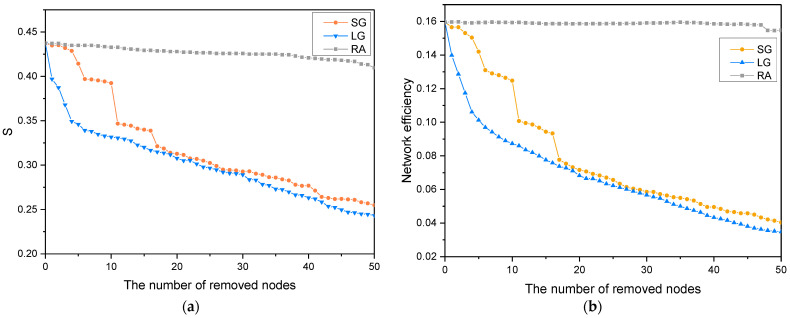
Comparative results of network robustness under successive node loss strategies. (**a**) changes in the relative size of the largest connected component; (**b**) changes in network efficiency.

Figure 4 shows that with the relative size of the LCC and the network efficiency in the SG and LG, node loss strategies decrease with an increase in the number of lost nodes in the network. In contrast, the change is small in the RA node loss strategy. This shows that the network’s robustness is significantly lower when facing the successive loss of structural hole nodes and opinion leader nodes than when facing random node loss. Furthermore, Figure 4a shows that the impact of the SG strategy on network robustness has obvious stages, which proves that there are project-based collaborative groups in the community, and structural hole nodes act as “bridges” to connect different groups. In the LG strategy, the network robustness is even worse. Here, when the top five opinion leaders are lost, network robustness drops by 20.1%, indicating that these five nodes are in the absolute core position within the network.

Figure 4b shows that the network has a higher network efficiency in the RA strategy, while the network efficiency decreases significantly in the SG and LG node loss strategies. Due to the small-world characteristics, the information transmission efficiency in the network is still high in the normal random loss strategy. However, when a few influential nodes are lost, the small-world characteristics of the network are destroyed and the connection performance of the network changes significantly. In the SG strategy, network efficiency shows a step—like downward trend. This is because the structural hole node is the “bridge” connecting the two network subgroups, and when it is removed, the information transmission channel between the two subgroups is cut off, disrupting the connectivity of the network. In the LG strategy, when the top opinion leader nodes are lost, network efficiency also drops sharply. Here, the decline rate is higher than that of the SG strategy, but it is continuous. Therefore, community managers should pay close attention to the project’s progress. When the project progresses slowly or stagnates, it may be that the top-ranked structural hole nodes and opinion leaders in the community are behaving abnormally, affecting the knowledge collaboration efficiency of users. Here, it is essential to formulate some incentive and protection mechanism for influential users.

### 5.3. Changes of Network Robustness When Considering the Following Effect

Opinion leaders in the OSPC transmit their viewpoints, ideas and other information to other users through knowledge collaboration, which gradually radiates to the whole network. In this process, user groups will worship, imitate and follow opinion leaders based on endogenous reasons. This is often called the “following effect”. The following effect describes the behavioral change of various individuals under group pressure, and it is often used to analyze the behavioral characteristics of a fixed group under specific conditions. In the internet environment, when facing a large amount of information, online users often follow the crowd, resulting in the emergence of the following effect. To study the impact of this on network robustness, we designed a strategy of loss of opinion leaders and their followers (RF), which represents the loss of opinion leaders in the community and the subsequent loss of some of their followers.

**Figure 5:**
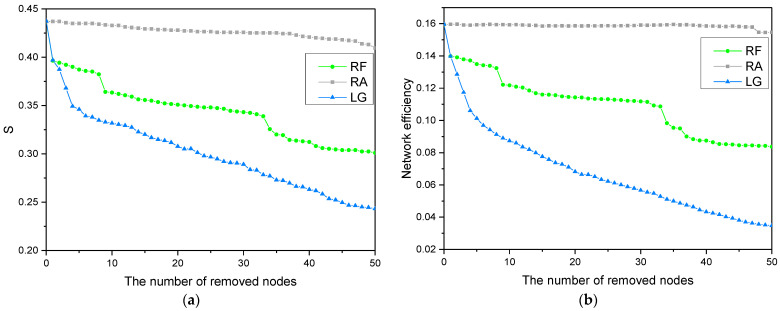
Changes in network robustness when considering the following effect: (**a**) changes in the relative size of the largest connected component; (**b**) changes in network efficiency.

This method involves removing the node with the strongest opinion leader ability in the current network, as well as the top ten follower nodes and edges (according to edge weight). The robustness indexes of the current network are then calculated. This is repeated n times to simulate the changes of network robustness when the nodes in the community follow the loss of opinion leader nodes. The simulation results are shown in Figure 5, which compares the robustness changes under the successive loss of opinion leaders.

Figure 5 shows that when removing the top opinion leader nodes in the network, the relative size of the LCC and network efficiency decline rapidly in the RF and LG strategies compared with the RA strategy. It shows that the robustness of the network in the RF strategy is lower than that in the RA strategy, but higher than that in the LG strategy, which demonstrates that there are many small user groups in the community. When some small groups are dissolved, it does not seriously damage the circulation of information in the network, which proves the advantages of the project-based and modular operation of the community. Further results show that when the most influential opinion leader node is removed, the relative size of the LCC decreases by 9.3% and the network efficiency decreases by 12.4%. When the followers of the opinion leader are removed, the relative size of the LCC and network efficiency continue to decrease, but the magnitudes of these decreases are reduced. When the seventh follower is removed, the relative size of the LCC and network efficiency decrease significantly again. Analyzing the attributes of the removed nodes shows that the seventh follower is also the opinion leader; therefore, the removal of this node has a great impact on the robustness of the network. This demonstrates that the identity characteristics of opinion leaders are not unique or fixed. With the increase of user participation and contribution in the community, some users will become opinion leaders in the community, while low-level opinion leaders have the desire to upgrade to higher-level opinion leaders. As such, community managers should formulate strategies to prevent the following effect when opinion leaders are lost. However, they should also recommend appropriate opinion leaders to more ordinary users, encourage them to actively participate in knowledge collaboration and to grow into influential users in the community as soon as possible.

## 6. Conclusions

The purpose of this paper was to explore the robustness of the open source product community (OSPC) network considering different influential nodes. First, based on the complex network, we analyzed the topological characteristics of the knowledge collaboration network of the OSPC and proposed an improved method based on the TOPSIS method and Burt’s structural hole theory to identify influential nodes. Then we proposed a variety of node loss strategies considering influential nodes and the following effect, and we constructed a simulation model to evaluate the impact of influential node loss on the robustness of the OSPC network. Our results indicate that the proposed method can better identify the influential nodes in the network, and that the robustness of the OSPC network under different node loss strategies shows different characteristics. Under a deliberate loss strategy, the loss of structural hole nodes in the network, either collectively or successively, significantly reduces the robustness of the knowledge collaboration network (KCN). Particularly, collective loss does great damage to the robustness of the network, indicating that the structural hole nodes acting as a “bridge” play an important role in the transmission of information in the network and the network structure. In addition, the loss of opinion leader nodes also significantly reduces the robustness of the KCN, which proves that opinion leaders have obvious influence in the community.

Our results also show that when the loss of opinion leaders in the community leads to the following effect, network robustness is reduced. However, this reduction is lower than the successive loss of opinion leaders, which shows that there are small groups relying on the project in the community due to the following effect. Thus, this structure enhances the robustness of the community to a certain extent.

To sum up, our research contributions are as follows:

First, a directed and weighted KCN model was established based on the knowledge dissemination and collaboration behavior among users in the real OSPC. Our study indicates that the KCN has obvious small-world and scale-free characteristics, and the behavior of users in the process of knowledge collaboration has greater heterogeneity. This provides support for analyzing the necessity of influential nodes in the network.

Second, most previous research on network robustness uses the degree and betweenness of nodes as the basis for node loss, paying less attention to the existence of many influential nodes (i.e., structural hole nodes and opinion leader nodes) [10,22,23]. This study explores the structural hole nodes in the network from the perspective of network structure characteristics, and evaluates the opinion leader nodes from the information dissemination perspective. Our study exposes these influential nodes as crucial to the dissemination of network information and knowledge in the open source product community (OSPC). The method proposed in this paper supplies a reference to identify critical nodes in complex networks.

Third, in the real community, the loss of users includes random natural loss and subjective deliberate loss. This study further subdivided the deliberate loss of influential users into collective loss and successful loss within a set period of time, and comprehensively explored the responses of the KCN to deliberate attacks. In addition, the influence of the following effect on the robustness of the KCN is studied, thus extending the research on network robustness. Our study also verifies the advantages of project-based and modular operation in the community, and discerns the dynamic of identified opinion leaders, which presents a new perspective for community managers to formulate management strategies.

Based on the analysis results, we obtained the following managerial implications:

First, the openness and technological progress of the internet have broken the traditional product design model. A large number of users have changed from passively receiving information to sharing information and creating new products through the OSPC. Ensuring the continuous participation of users and attracting more users to join the community is the key to the sustainable operation of the community, as well as the core challenge for managers in building a prosperous community. Although some studies have shown that managers should pay attention to the main participants in the community, our research shows that they should pay more attention to the influential users in the community. Furthermore, a sound community management mechanism is fundamental to the good development of the community [8,9]. As such, we cautiously recommend that the community establishes priority protection mechanisms for its influential users (e.g., mechanisms to prevent malicious harassment, protect private information and intellectual property, etc.). It also suggests that the community should formulate personalized incentive strategies for these users, publicizing their contributions and achievements, and giving them material and non-material rewards. Such affirmations can improve influential user loyalty to the community.

Second, the following effect can focus users and promote the faster completion of a project [34]. In OSPCs, this following effect is often ignored by community managers. Our results demonstrate that when the following effect occurs, the robustness of the network is also greatly damaged. Therefore, to prevent users from blindly following the trend (such as when opinion leaders leave the community), it is necessary to establish a guidance mechanism for users in the community. At the same time, increasing the diversification of projects in the community and establishing a project recommendation mechanism based on user attributes will also be beneficial to the development of the project.

Third, the contributions and roles of users to the community are dynamic [8]. Our results highlight the relationship between opinion leaders and users occupying structural holes. Therefore, community managers should fully utilize the characteristics and attributes of influential users, formulating incentive strategies for structural hole users, to promote their rapid growth into opinion leaders. Meanwhile, the community should also develop diversified incentive strategies for its ordinary users, encouraging them to follow the example set by influential users, to improve their own contribution. Due to the dynamic evolution of the OSPC, the contribution and importance of users are not invariable. In future research, we will explore the transformation mechanism between ordinary nodes, structural hole nodes and opinion leader nodes. Further, we will study the impact of user identity transformation on the robustness of the network. This will provide suggestions for community managers to effectively develop users, optimize the community structure and improve the efficiency of knowledge dissemination.

## Figures and Tables

**Table 1:** Topology parameters and network characteristics of the knowledge collaboration network.

Topology Parameters	Number of Nodes	Average Degree	Average Path Length	Clustering Coefficient	Average Proximity Centrality	Average Betweenness Centrality	Small-World Characteristic
	1428	6.27	3.04	0.53	1,322,627.3	1278.6	155.89
Network Characteristic	Small-world Characteristic		Scale-free Property		Assortativity		
	Yes		Yes		heterogeneous network		

Note: According to Davis et al. [40], small-world parameter SW = [Cactual/Lactual] ∗ [Lrandom/Crandom]; Therefore, Lrandom = ln (n)/ln (k), Crandom = k/n, where n is the number of nodes and k is the average degree.

**Table 2:** List of top ten nodes based on constraint degree index.

Node Number	Degree of Constraint	Effective Scale	Efficiency	Degree of Hierarchy
26	0.031	300.262	0.944	0.406
899	0.032	91.096	0.969	0.167
142	0.033	249.065	0.933	0.369
77	0.039	179.164	0.909	0.325
149	0.040	311.546	0.953	0.539
130	0.041	283.231	0.947	0.531
7	0.046	130.875	0.909	0.292
118	0.048	101.88	0.842	0.197
649	0.050	94.694	0.831	0.203
43	0.052	106.188	0.885	0.251

**Table 3:** Top ten opinion leaders of the knowledge collaboration network.

Node Number	711	19	149	130	26	142	7	77	649	118
$O_{i}$	0.959	0.661	0.621	0.606	0.530	0.468	0.285	0.281	0.203	0.168

**Table 4:** Node loss strategies and their practical significance.

Node Loss Type	Node Loss Strategy	Strategy Description	Practical Significance
Random node loss	Random node loss (RA)	Randomly select n nodes for removal to simulate the irregular loss of users.	The irregular loss of nodes.
Deliberate node loss	Collective loss of structural hole nodes (SC)	Sort the structural hole nodes in the initial network, then select and remove the top n nodes to simulate the collective loss of users who occupy the structural hole location in the community.	The collective loss of users who adopt the “bridging” role in the community.
	Collective loss of opinion leader nodes (LC)	Sort the opinion leader nodes in the initial network, then select the top n nodes and remove them to simulate the collective loss of opinion leaders in the community.	The collective loss of opinion leaders in the community.
	Successive loss of structural hole nodes (SG)	Sort the structural hole nodes in the initial network, then remove the node with the highest structural hole index value. Remove the edges associated with the leaving node. Calculate the robustness of the network at this time. Recalculate the structural hole index value of the nodes in the current network, and sort them in descending order. Repeat the above steps n times to simulate the successive loss of n users who occupy the structural hole location in the community.	The successive loss of users who play the role of “bridge” in the community
	Successive loss of opinion leader nodes (LG)	Sort the opinion leader nodes in the initial network, then remove the node with the highest opinion leader index value. Remove the edges associated with the leaving node. Calculate the robustness of the network at this time. Recalculate the opinion leader index value of the nodes in the current network, and sort them in descending order. Repeat the above steps n times to simulate the successive loss of n opinion leader users in the community.	The successive loss of opinion leaders in the community.

## Data Availability

The used and analyzed datasets during the present study are available from the corresponding author on reasonable request.

## Abbreviations

The abbreviations used in this manuscript are as follows:

OSPC	Open source product community
OSD	Open source design
OSCs	Open source communities
KCN	Knowledge collaboration network
RA	Random node loss
SC	Collective loss of structural hole nodes
LC	Collective loss of opinion leader nodes
SG	Successive loss of structural hole nodes
LG	Successive loss of opinion leader nodes
LCC	Largest connected component
RF	The strategy of loss of opinion leaders and their followers
